# Photocatalytic hydrogen evolution from biomass conversion

**DOI:** 10.1186/s40580-021-00256-9

**Published:** 2021-02-26

**Authors:** Kayla Alicia Davis, Sunghoon Yoo, Eric W. Shuler, Benjamin D. Sherman, Seunghyun Lee, Gyu Leem

**Affiliations:** 1 Department of Chemistry, State University of New York College of Environmental Science and Forestry, 1 Forestry Drive, Syracuse, NY 13210 USA; 2 Department of Chemistry, Gachon University, Seongnam, Gyeonggi-do 13306 Republic of Korea; 3 Department of Chemical and Molecular Engineering, Hanyang University, Ansan, Gyeonggi-do 15588 Republic of Korea; 4 Department of Chemistry and Biochemistry, Texas Christian University, Campus Box 298860, Fort Worth, TX 76129 USA; 5 The Michael M. Szwarc Polymer Research Institute, 1 Forestry Drive, Syracuse, NY 13210 USA

**Keywords:** Hydrogen production, Biomass, Photocatalysis, Energy and charge transport, Solar energy conversion

## Abstract

Biomass has incredible potential as an alternative to fossil fuels for energy production that is sustainable for the future of humanity. Hydrogen evolution from photocatalytic biomass conversion not only produces valuable carbon-free energy in the form of molecular hydrogen but also provides an avenue of production for industrially relevant biomass products. This photocatalytic conversion can be realized with efficient, sustainable reaction materials (biomass) and inexhaustible sunlight as the only energy inputs. Reported herein is a general strategy and mechanism for photocatalytic hydrogen evolution from biomass and biomass-derived substrates (including ethanol, glycerol, formic acid, glucose, and polysaccharides). Recent advancements in the synthesis and fundamental physical/mechanistic studies of novel photocatalysts for hydrogen evolution from biomass conversion are summarized. Also summarized are recent advancements in hydrogen evolution efficiency regarding biomass and biomass-derived substrates. Special emphasis is given to methods that utilize unprocessed biomass as a substrate or synthetic photocatalyst material, as the development of such will incur greater benefits towards a sustainable route for the evolution of hydrogen and production of chemical feedstocks.

## Introduction

The global annual demand for hydrogen gas as a viable alternative fuel has increased in the global energy system, mainly from traditional uses such as ammonia production and metal refinery industries, but more increasingly from the development of hydrogen fuel cells, a key part of a clean-energy future for humanity [1]. The production of hydrogen gas for use in hydrogen fuel cells represents an effective and carbon free strategy for the chemical storage of energy. The oxidation of H_2_ coupled to the reduction of O_2_ in a fuel cell produces water as the only product, a more environmentally friendly process than fossil fuel combustion, which produces harmful greenhouse gas emissions in the form of CO_2_. Moreover, hydrogen is also attractive as an alternative energy source because of its high energy density (e.g., 141.8 ⨯ 10^6^ kJ/kg), which is greater than that of most fuels (e.g., 44 ⨯ 10^6^ kJ/kg for gasoline) at room temperature [2]. The most prevalent production method for H_2_ is steam reforming of methane feedstocks, producing nearly half of the hydrogen gas in the world [3]. However, this method requires substantial energy input (e.g. > 750 °C), whereas an ideal method of hydrogen gas production would utilize renewable resources as feedstocks and energy inputs [4]. Using solar energy to photochemically produce H_2_ from biomass presents an attractive alternative to the energy demanding natural gas reformation process. Photoreforming of biomass is a promising method of hydrogen evolution, not only because the method relies on foreseeably infinite solar energy inputs, but also because it relies on renewable biomass substrate and could utilize the byproducts from existing industrial biomass processes, e.g. ethanol from sugar fermentation [5]. Moreover, biomass photoreforming systems have been developed for hydrogen evolution that utilize catalysts or electrodes that are synthesized from biomass utilization byproducts or biomass itself [4, 6–9]. For example, Han et al*.* reported that photocatalytic oxidation of biomass intermediates was integrated with H_2_ production in aqueous media, and chemical transformation of biomass intermediates (e.g., 5-hydroxymethylfurfural, HMF) to value-added products (e.g., aldehydes and acids) was achieved [6].

**Fig. 1:**
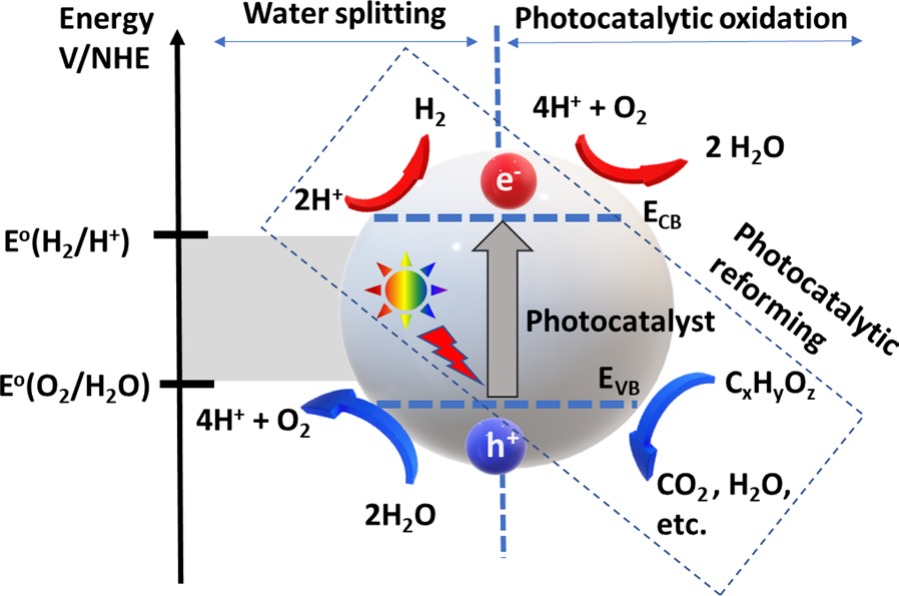
Schematic illustration of water splitting and photocatalytic oxidation of biomass over photocatalyst nanoparticle under light illumination. (Adapted from Kondarides et al. and Huang et al. [9, 10])

Hydrogen production from photocatalytic water splitting (without biomass) is achievable, but at a very low quantum yield (~ 1.8%) due to a thermodynamic barrier and multi-electron transfer process [11], while quantum yield of photobiorefinary for H_2_ production is achievable above 70% [12]. The photocatalytic reforming of biomass and biomass-derived substrates is energetically comparable to the overall water splitting process, except biomass replaces water as the electron and proton source, as shown in Fig. 1 [9, 10, 13]. The electronic band structures and energies of photocatalysts are crucial in the photophysical events of electron/hole transport and charge carrier injection for water splitting or the production of H_2_ from biomass. By illuminating a photoactive semiconducting material or aqueous suspension, an electron–hole pair separation occurs when an electron from the valence band of the semiconducting material is excited into the conduction band, leaving behind a photo-generated hole. The capacity of the photogenerated charge carrying species (the electron–hole pair) to facilitate a redox reaction (reducing hydrogen and oxidizing biomass) depends on the potential of the semiconductor’s conduction/valence band in reference to the reduction/oxidation potential of the redox species in question, i.e. water. The favorable conduction/valence band levels of titania and related semiconducting materials in respect to the redox process of forming H_2_ from biomass has been demonstrated [10, 13]. Photo-generated holes in titania can oxidize substrate in the solution, i.e. water, biomass, or biomass-derived substrates, thereby generating protons, carbon dioxide, and reactive radicals through extractive single-electron transfer. The photo-excited electrons, conversely, reduce protons in solution to generate hydrogen gas. Without available substrates capable of reduction/oxidation interactions with the charge-carrying photogenerated electron–hole pairs, the promoted electron would migrate back to the valence band by releasing thermal energy, recombining with the hole [13]. The biomass oxidation reaction and hydrogen evolution reaction can concomitantly oxygenate electron-donating biomass and biomass-derived substrates and reduce protons into molecular hydrogen, albeit not at the same rate. The use of cocatalysts to expand the absorption capabilities of the semiconducting materials or to provide efficient proton reduction sites is required for ideal hydrogen production rates [8].

The conversion of visible light irradiation (e.g. solar light) into hydrogen can provide an important industrial method of hydrogen gas production without costly and intensive energy input. The aim of this manuscript is to identify possible avenues by which the photocatalytic production of hydrogen can become industrially feasible. By avoiding methods that require high energy input and that rely on fossil fuels, the scale-up of these reactions should be feasible, especially considering the use of cheap and sustainable biomass feedstocks that specifically do not compete with the food industry, i.e. non-edible biomass such as crop and forest residuals or animal wastes. Using earth-abundant semiconductor materials as photocatalysts to sustain solar-to-hydrogen conversion of non-edible biomass-derived substrates and water is of particular interest. Other key factors to consider for the reformation of biomass to H_2_ include the overall reaction conditions; not just the semiconducting materials used, but also the use of co-catalysts, the presence of oxygen, and the solution identity, etc. Particularly when reaction conditions are ambient, greater control over the conversion of substrate functional groups (or avoidance of side-reactions) is possible, while energy inputs remain industrially relevant. In this review, the visible light-induced photocatalytic reforming process of biomass is mainly categorized and outlined by the photocatalyst type (metal vs. metal-free) and the reforming substrate (monomeric, polymeric, or raw biomass). Photocatalytic mechanisms and the key characteristics of heterogeneous photocatalysts and reforming substrates for solar or visible light driven renewable biomass conversion to hydrogen are considered.

## Photocatalysis and mechanisms

### TiO_2_-based photocatalysts and cocatalyst loading

Since titanium dioxide (TiO_2_) was discovered to be effective in photoelectrochemical (PEC) water splitting in the early 70′s [14], extensive research has been conducted on TiO_2_ as the most used photocatalyst for hydrogen generation [13, 15]. However, its main drawbacks are a large bandgap of 3.2 eV, low electron mobility, and short diffusion length [16]. A main disadvantage of TiO_2_ is that it only absorbs in the UV range (λ < 387 nm) and the solar spectrum is relatively weak in this range of wavelength (~ 4%) [17]. Because of these photocatalytic pitfalls, loading with metal (e.g., platinum) and nonmetal cocatalysts, and modification with visible-light sensitizers (e.g., CdS, g-C_3_N_4_, Eosin Y) has been widely applied to enhance the photocatalytic activity of TiO_2_ for the hydrogen production from water and biomass derivatives [13, 18–21].

### *Pt/TiO*_*2*_

**Fig. 2:**
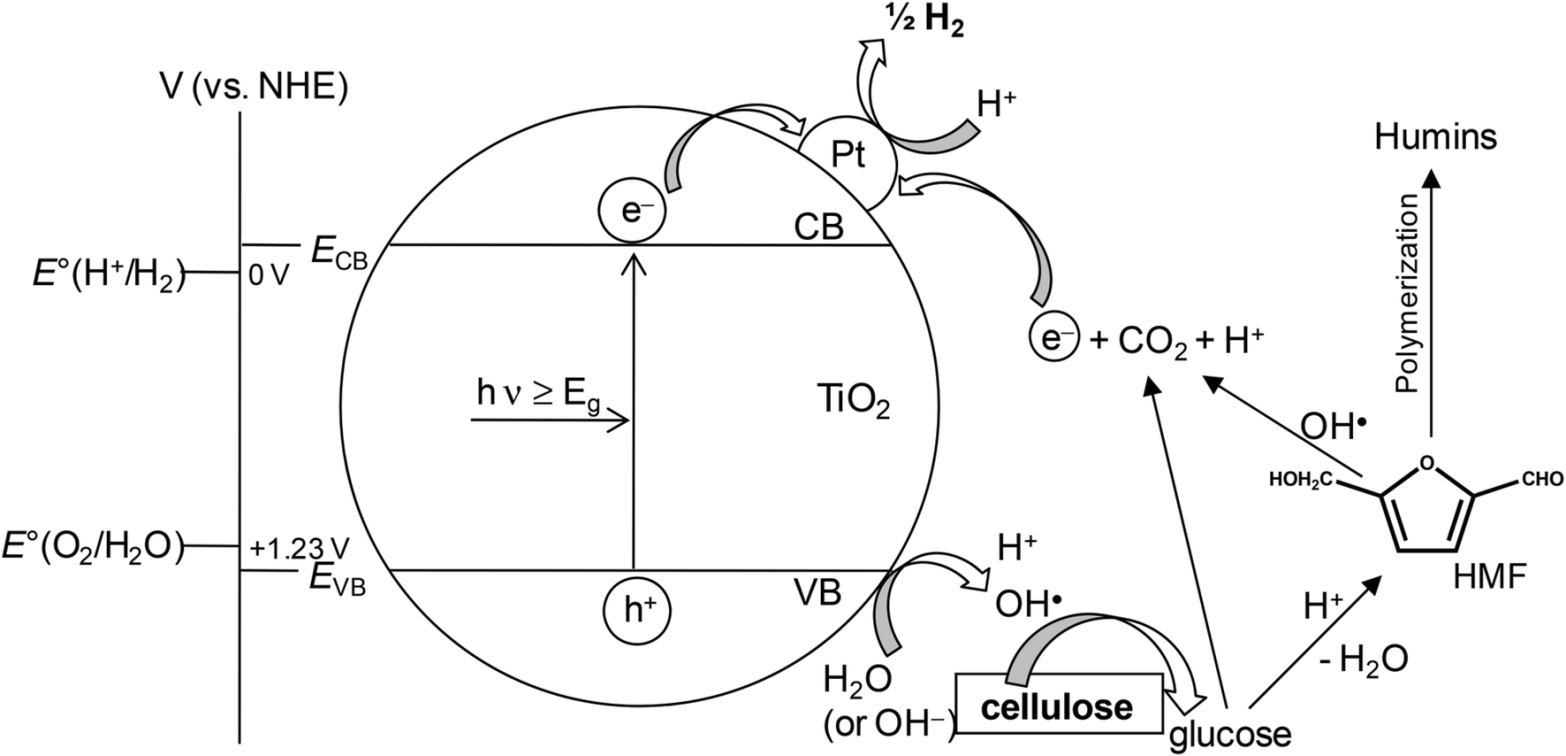
Schematic illustration of the photocatalytic H_2_ evolution using Pt/TiO_2_ in the presence of cellulose. Reprinted with permission from Speltini et al. [17] (Copyright 2014 Royal Society Chemistry (RSC). Published by the RSC on behalf of the European Society for Photobiology, the European Photochemistry Association, and RSC)

Doping with Pt (Pt/TiO_2_) effectively improves photocatalytic hydrogen evolution because Pt is a robust proton reduction catalyst; the increased rates of H_2_ formation of Pt/TiO_2_ under illumination are evidence of the suppression of electron–hole recombination [18, 22, 23]. For example, an investigative study of reaction conditions for hydrogen evolution from cellulose with Pt/TiO_2_ under visible light illumination was performed as shown in Fig. 2 [17]. This general scheme shows the main reaction steps combining visible light-induced water splitting and biomass oxidation in the presence of cellulose. Cellulose acts as a sacrificial reducing agent and combines with the photo-generated oxidant species which suppresses charge carrier recombination as shown in Fig. 2. This process is necessary to improve H_2_ production [24]. Moreover, the hole and oxidant species (e.g., ·OH radicals) activated the depolymerization of cellulose to form glucose products. Glucose is one of the most efficient sacrificial mediators for photocatalytic H_2_ production [24]. Also, this work demonstrated that neutral pH was optimal for hydrogen production and that minerals found in seawater did not affect the production rate. Moreover, the formation of 5-hydroxymethylfurfural (HMF) oligomers during photocatalytic production, formed by the further oxidation of glucose, results in an “in situ dye-sensitization”, where their adsorption onto the TiO_2_ photocatalyst surface enhances hydrogen production by promoting light capture and charge transfer, either to the Pt catalyst or to the TiO_2_ conduction band through photoexcited electron injection. When using alfalfa and rice husk biomass as feedstocks, it is recognized that lignin does not interfere with the photocatalytic hydrogen production as an inhibitor or as a radical scavenger.

The use of Pt/TiO_2_ catalyst in sulfuric acid media combined acid hydrolysis and photocatalysis to photoreform cellulose to hydrogen and HMF (from glucose dehydration), where the H_2_ yield and the glucose yield were 66% and 85% respectively [25]. This result suggests that the photocatalytic formation of hydrogen is the rate-limiting step as opposed to cellulose hydrolysis if loading is maximized. It was demonstrated that H_2_ production is five-fold greater when coupled with acid hydrolysis. A hydrogen production rate of 1320 μmol h^−1^ g_cat_^−1^ was achievable in the same system with raw biomass paper pulp. α-Cellulose decomposition to sugars, carbon dioxide, and hydrogen under UV/solar radiation by aqueous suspension of cellulose anchored onto Pt/TiO_2_ was investigated [26]. Immobilization of cellulose onto a Pt/TiO_2_ photocatalyst and the presence of Pt were crucial for enhancing the conversion efficiency. Although, an increased ratio of immobilized cellulose reduced the rate of cellulose conversion, most likely due to the increased difficulty in oxidation of the small, soluble cellulose-degradation products by diffusion-limited OH radicals. The main products of cellulose degradation in this method were glucose, cellobiose, and formic acid. After seven cycles, the H_2_ production yield was 80–90%, while the CO_2_ production yield was 70–80%. The surface-bound cellulose in the whole process plays an important role as the sacrificial agent to boost the H_2_ production yield.

### *Pd/TiO*_*2*_

**Fig. 3:**
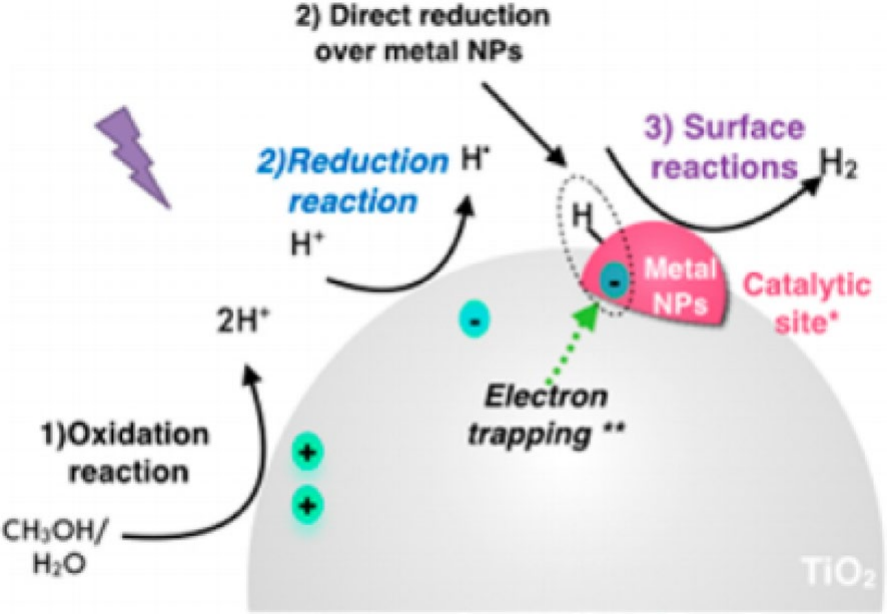
Schematic illustration of the photocatalytic H_2_ evolution using Pt/TiO_2_ in the presence of CH_3_OH and H_2_O. Reprinted with permission from Luna et al. [28] (Copyright 2017 American Chemical Society)

Pt is the most efficient and effective co-catalyst for hydrogen generation when coupled to TiO_2_ [27]. Due to the high cost and limited availability of Pt, Pd represents a viable alternative because of its lower cost and greater abundance than Pt [28]. Pd doped TiO_2_ (Pd/TiO_2_) has been actively investigated in photocatalysis especially for water splitting and biomass reforming [16, 28–32]. Luna et al*.* reported a high photocatalytic activity for H_2_ generation using a TiO_2_ surface modified with Pd nanoparticles prepared by radiolysis [28]. Radiolysis is a powerful technique to synthesize metal nanoparticles and control the morphology in solutions [31]. Three steps, (1) light absorption, (2) the charge-carrier dynamics, and (3) surface reactions, were mainly involved in this photocatalytic H_2_ generation over Pd/TiO_2_. In Fig. 3, atomic hydrogen (H·) is formed by the reduction reaction of H^+^ (Eq. 1) on the TiO_2_ surface from water splitting or alcohol oxidation. Then, atomic hydrogen (2H·) combines to give molecular hydrogen or reacts with alcohol molecules on the Pd or Pd/TiO_2_ surface (Eqs. 2, 3). The surface-bound Pd nanoparticles serve as catalytic sites to facilitate the enhanced H_2_ evolution. In other words, the hydrogen production is mainly controlled by the surface-bound Pd nanoparticles.

1 $${\text{H}}^{ + } + {\text{ e}}^{ - } \to {\text{ H}} \cdot$$

2 $${\text{H}} \cdot + {\text{H}} \cdot \to {\text{ H}}_{{2}}$$

3 $${\text{H}} \cdot + {\text{ CH}}_{{3}} {\text{OH }} \to {\text{ H}}_{{2}} + {\text{CH}}_{{2}} {\text{OH}} \cdot$$

### *Au/TiO*_*2*_

The *in-situ* photodeposition of Au metal onto a TiO_2_ surface has been explored for the photocatalytic hydrogen evolution from biomass solution [33–38]. For example, Gomathisankar et al*.* reported hydrogen evolution from glucose (representative biomass) performed with Au/TiO_2_ photocatalysts [33]. Under illumination, photogenerated electrons in the TiO_2_ drive the surface reduction of Au^3+^ ions to Au(0). Then, the Fermi levels of the interface of Au-TiO_2_ are aligned. This process minimizes electron–hole recombination which facilitates photocatalytic hydrogen production. The holes are quenched by glucose molecules as sacrificial electron donors. Holes continuously react with H_2_O and glucose to produce protons, hydroxyl radical species, CO, and CO_2_. During this degradation process of glucose over Au/TiO_2_, gluconic acid, glucaric acid, and arabitol were obtained. Interestingly, under visible light irradiation, the surface plasmon resonance (SPR) effect in the presence of Au metal results in an increase in the density of photo-excited electrons in the TiO_2_. As a result, photocatalytic activity for hydrogen production under LED light illumination (365 nm lamp) increased by 203 times compared to the activity of bare TiO_2_.

The Fu research group explored photocatalytic reforming of glucose over Au/TiO_2_ photocatalyst under a 125 W high-pressure mercury lamp and observed hydrogen generation at a rate of 1.37 mmol h^−1^ [39]. Interestingly, under simulated solar light illumination, the hydrogen production yield increases to 5–6 mmol g^−1^ h^−1^ with high selectivity (> 99.3%) for H_2_ product by ethanol photoreforming on Au/TiO_2_ solids prepared by the deposition–precipitation method [35]. On the basis of these results, pure H_2_ can be generated from cheap biomass-derived feedstock such as ethanol. Similarly, Murdoch et al*.* reported photocatalytic H_2_ production from ethanol using Au/TiO_2_ [36]. Au was loaded on both the anatase and rutile TiO_2_ nanoparticles. The rate of H_2_ production from ethanol over Au on anatase TiO_2_ nanoparticles was two orders of magnitude higher than that with Au on rutile TiO_2_ nanoparticles due to the higher Fermi level of anatase resulting in greater electronic interaction with Au.

**Fig. 4:**
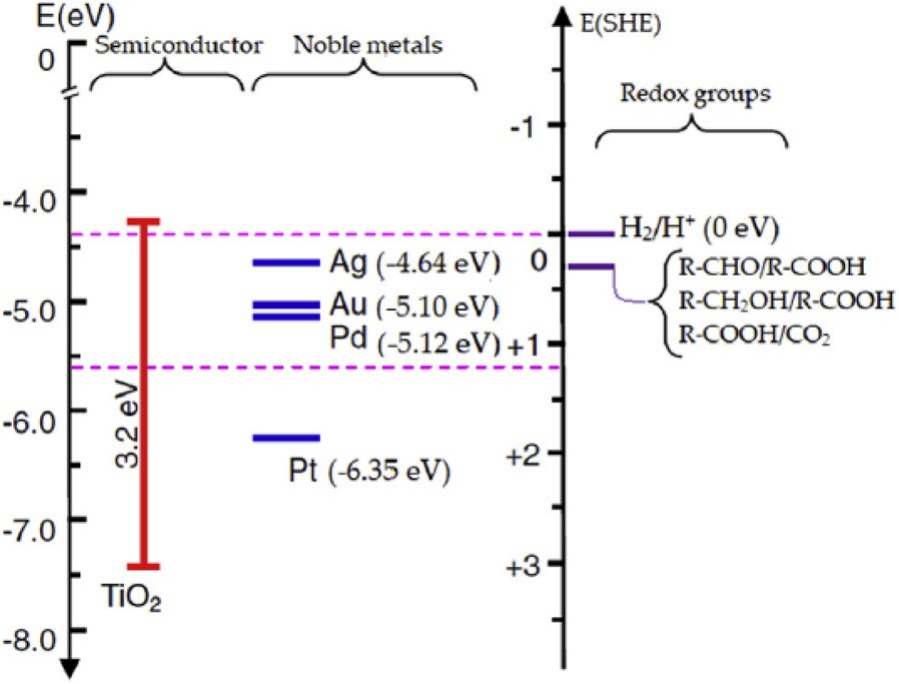
Energy band positions, the work functions of the noble metals, and the electrochemical potentials of the redox couples involved. Reprinted with permission from Ramis et al. [40] (Copyright 2020 Elsevier)

Ramis et al*.* studied the use of other noble metal co-catalysts on TiO_2_ including Au [40]. TiO_2_ with Pt and Pd exhibited a higher photocatalytic activity than that with Au. The metal work functions of Au, Pd, and Pt are 5.10, 5.12, and 6.35 eV, respectively (Fig. 4). The smaller Schottky barrier formed between TiO_2_ and Au allows increasing rates of recombination for photogenerated electronic-hole pairs resulting in suppressed photocatalytic activity.

### *NiS/TiO*_*2*_

**Fig. 5:**
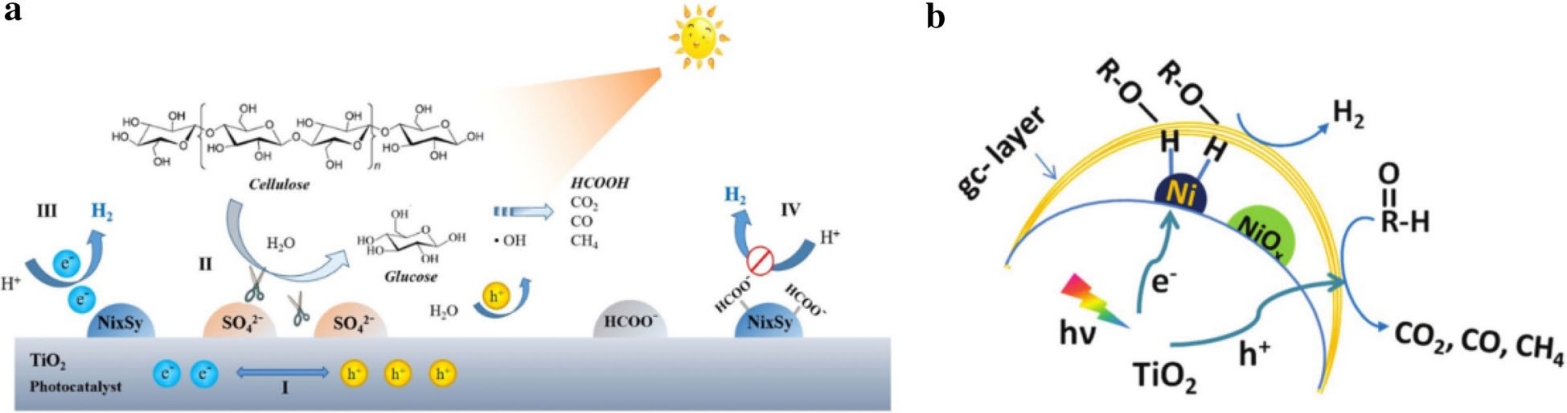
Proposed mechanism of cellulose photoreformation into H_2_ over **a** NiS/TiO_2_ and **b** NiOx/TiO_2_. Reprinted with permission from Hao et al. [41] and Zhang et al. [42] (Copyright 2018 ChemPubSoc Europe and Royal Society Chemistry)

To avoid the use of expensive noble metal co-catalysts, Hao and co-workers reported a system with TiO_2_ semiconductor particles modified with nickel sulfide and sulfate for the photocatalytic reformation of cellulose to hydrogen (Fig. 5a) [41]. Interestingly, the modification of TiO_2_ with NiS (NiS/TiO_2_) demonstrated a similar hydrogen production rate as compared to Pt/TiO_2_. The coordination bound sulfate species assists in the dissolution of biomass and in the hydrolysis of cellulose; however, formate species were found to poison the catalyst through the occupation of active sites, with increasing effect as the reaction progressed. The desorption of formate can be controlled by the alkalinity of the system but at the sacrifice of the hydrogen production efficiency.

### *NiO*_*x*_*/TiO*_*2*_

NiO_x_/TiO_2_ NP loaded with a graphite carbon layer was reported to produce 270 μmol h^−1^ g^−1^_cat_ of hydrogen at room temperature, although this rate increased to 4150 μmol h^−1^ g^−1^_cat_ when the reaction temperature was raised to 80 °C, purportedly due to the increased solubility of carbohydrate byproducts [42]. An interface of NiO_x_ with a graphitic overlayer was required to improve biomass photoreforming activity. Proton reduction occurs between the carbon layer and NiO_x_/reduced Ni sites (Fig. 5b). The carbon layer served to weaken the O–H bond of the alcohol or saccharide substrate and reduced Ni sites afforded a high rate of H_2_ evolution from the NiO_x_/TiO_2_ surface.

### *SnO*_*x*_*-RuO*_*2*_*/TiO*_*2*_

Anatase TiO_2_ grafted with single-site SnO_x_ and loaded with RuO_2_ is reported as a photocatalyst for hydrogen evolution from various biomass species C_x_H_y_O_z_ [43]. The interfacial Ti(IV)–O–Sn(IV) for electron transfer and Ti(IV)–O–Ru(IV) for hole transfer linkages facilitate efficient charge separation, improving the production of hydrogen from water splitting which is optimized at 0.68 wt% RuO_2_ and 0.24 wt% Sn species. The modification of TiO_2_ with metal oxide co-catalysts plays an important role by enhancing the proton reduction reaction from the photocatalytic reforming of biomass-derived molecules.

### CdS-based photocatalysts and cocatalyst loading

CdS nanomaterials are widely used as photosensitizers or photocatalysts because of their excellent photophysical properties such as a high molar extinction coefficient in the visible region [44]. Despite this property, high charge recombination followed by the photoexcited CdS can result in limited use as a photocatalyst for H_2_ evolution from biomass and biomass-derived substrates [45].

#### Au/CdS Nanorods

**Fig. 6:**
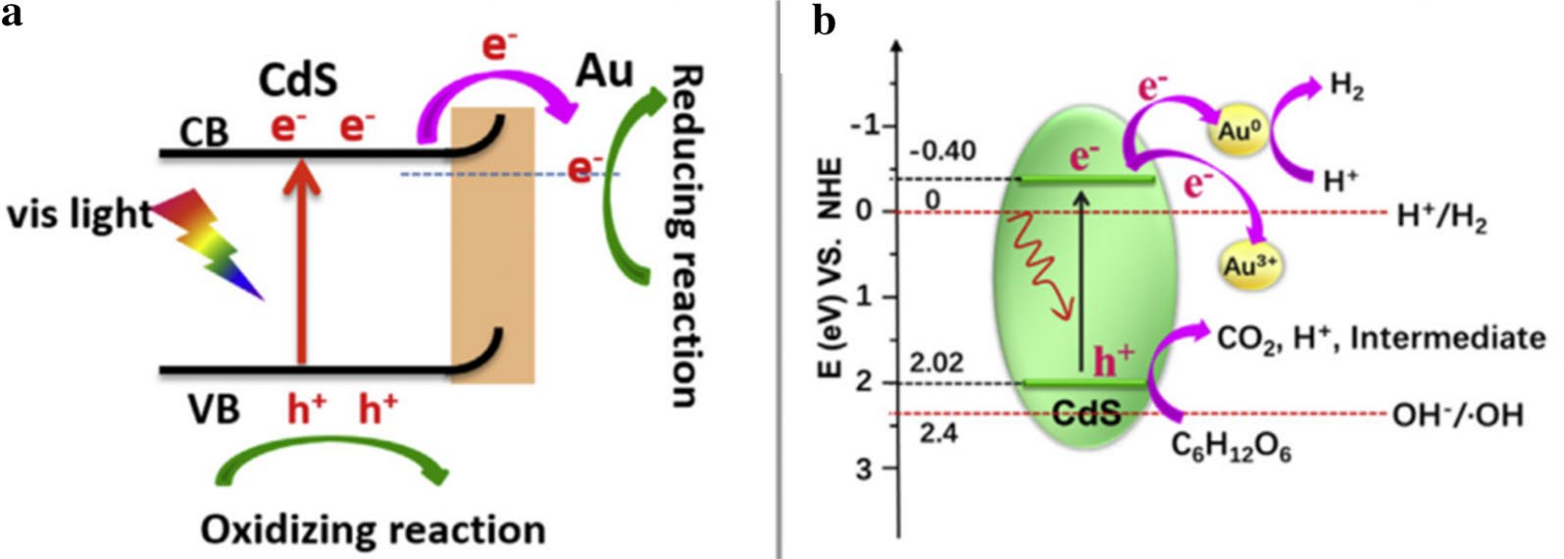
The schematic band diagram (**a**) and proposed mechanism (**b**) for photocatalytic hydrogen production with Au/CdS NRs in glucose solution under visible light irradiation. Reprinted with the permission from Wang et al. [8] (Copyright 2020 Elsevier)

Wang and co-workers reported Au^3+^ doped on the surface of CdS nanorods (Au/CdS-NRs) to solve this fundamental issue of high charge recombination [8]. 1 D CdS-NRs have unique properties including high photon absorption cross-sections and optical gain lifetimes and relatively strong permanent electric dipoles compared to spherical nanocrystals of CdS [46]. As shown in Fig. 6, Au^3+^ can be easily reduced to Au^0^ by the photogenerated electrons from photoexcitation of Au/CdS compared to the reduction of H^+^ to H_2_ [8, 47]. These self-reduced Au nanoparticles can increase the charge transfer leading to better separation of electron–hole pairs. Upon photoexcited electron transfer, the holes are left on the valence band of CdS nanorods. These resulting holes can oxidize glucose. Therefore, self-reduction of Au can suppress the charge recombination process resulting in the enhanced photocatalytic efficiency for H_2_ evolution from oxidized glucose under visible light illumination. This work inspired a pathway to construct heterojunction photocatalysts for the H_2_ evolution from biomass.

#### Ni/CdS nanosheets

**Fig. 7:**
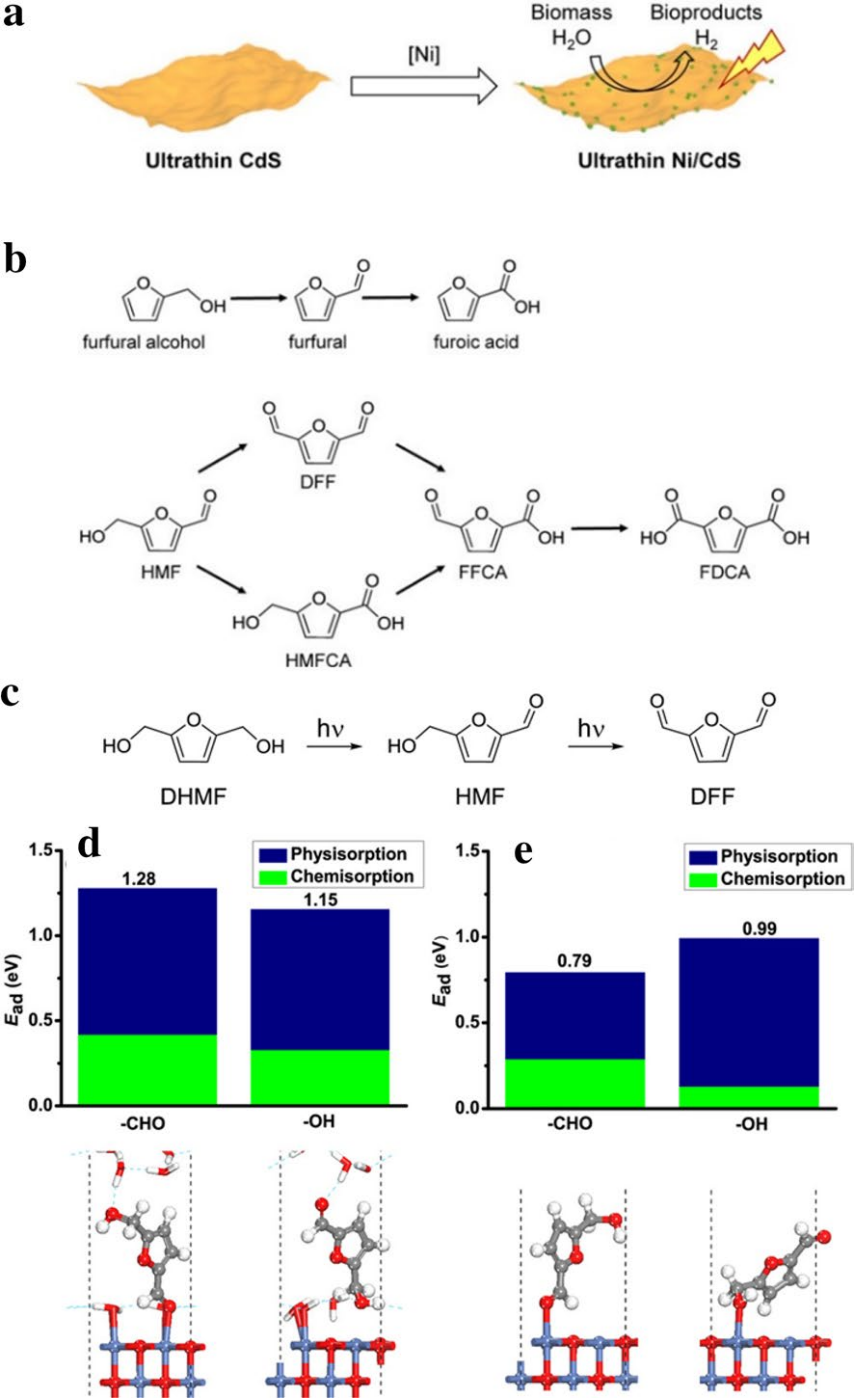
**a** The illustration of the synthetic route of Ni/CdS nanosheets. **b** Oxidation reactions of the alcohol and aldehyde groups in HMF. **c** Oxidation of DHMF to HMF and DFF. Adsorption structures and energies of MHF **d** at water/NiO interface and **e** over bare NiO surface vias its aldehyde or alcohol group. Reprinted with the permission from Han et al. [6] (Copyright 2017 American Chemical Society)

Minimizing charge recombination of excited electrons and holes within the light absorbing semiconductor, especially for CdS, is critical to enhancing H_2_ yield from biomass because this process competes with proton reduction to H_2_. General strategies employed to avoid recombination include the use of sacrificial electron donors; however, these strategies can reduce the oxidizing capability of the excited holes. Instead of utilizing sacrificial agents as hole scavengers, CdS nanosheets loaded with nickel co-catalyst (CdS/Ni) can suppress charge recombination and drive organic transformation resulting in H_2_ production and the formation of value-added chemicals from biomass conversion [6]. As shown in Fig. 7a, Ni was deposited to ultrathin CdS nanosheets. Then, the biomass-derived intermediate compound, 5-hydroxymethylfurfural, HMF, was converted to value-added bioproducts (e.g., 2,5-diformylfuran, DFF) and H_2_ under a photocatalytic Ni/CdS system in aquatic media with a blue LED (450 nm, 8 W) (Fig. 7b). Photo-formed holes were theorized to migrate to the nickel oxide sites after excitation, suppressing electron–hole pair recombination while also charging these sites for biomass oxidation. HMF preferentially absorbs to the Ni sites, leading to the photocatalytic reaction at the interface. Moreover, the oxidative photocatalytic conversion of HMF to DFF (biomass intermediate) was investigated as shown in Fig. 7c. Interestingly, this oxidation of HMF containing one alcohol and aldehyde moiety exhibited a lower conversion than the oxidation of furfural alcohol (with monoalcohol group). According to theoretical computation calculations, the aldehyde moiety in HMF prefers to vertically adsorb atop the water/Ni site and binds more strongly than the alcohol group in HMF (Fig. 7d). As a result, the alcohol group is far away from the NiO surface, which disfavors alcohol oxidation. Therefore, during the photocatalysis reaction, slow oxidation from HMF to DFF occurs under neutral water conditions. It is interesting to note that HMF prefers to be bound to NiO surface via its alcohol moiety rather than aldehyde once in the absence of water (Fig. 7e).

#### NiS/CdS

Nickel sulfide (NiS) as a p-type semiconductor has been used for photocatalytic H_2_ production when incorporated with TiO_2_ or g-C_3_N_4_ [48–50]. Li and co-workers reported one-dimensional NiS/CdS nanostructures for visible light-driven H_2_ evolution from lignocellulose biomass, lactic acid, and lignin [50]. The presence of the NiS can enhance charge separation and charge transfer at the interfaces between CdS and NiS. Moreover, lactic acid and lignin were used as the hole scavengers that led to efficient charge separation in CdS. Thus, the photocatalytic hydrogen production yield with the NiS/CdS nanocomposites from lignin and lactic acid was substantially increased compared to CdS.

**Fig. 8:**
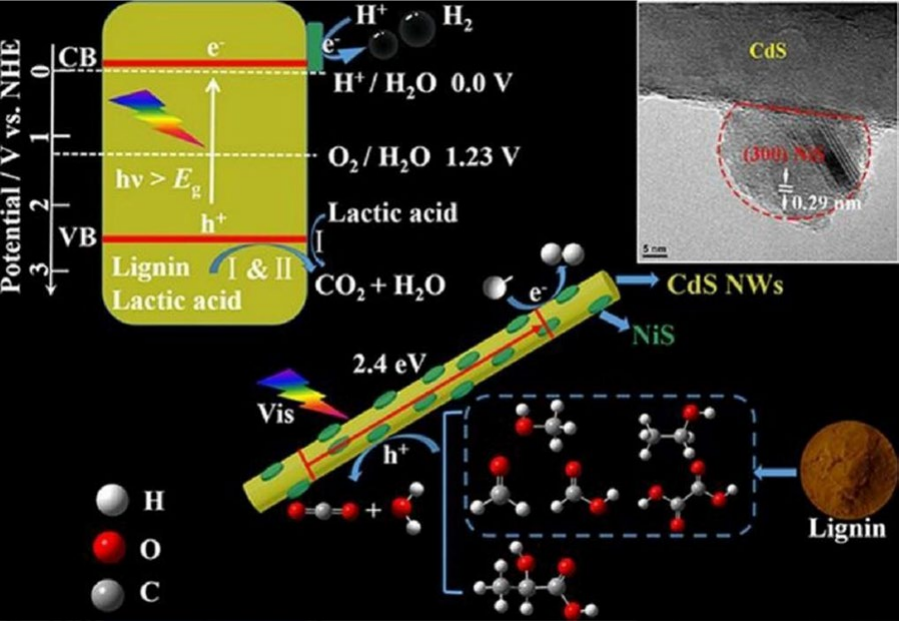
**a** The schematic illustration of the photocatalytic hydrogen production with lignin and lactic acid over NiS/CdS nanocomposites under visible light illumination. Reprinted with the permission from Li et al. [50] (Copyright 2018 Elsevier)

The proposed mechanism of photocatalytic H_2_ production is shown in Fig. 8. The conduction band (CB) of surface-attached NiS is less than that of CdS. Upon the photoexcited CdS, the resulting photogenerated electrons can be injected into the CB of NiS. Then, the activated NiS strongly attracts H^+^ in the solution due to the unsaturated sulfide ions of NiS. This strong affinity for H + can boost the photocatalytic reduction of H^+^ to H_2_. The photoinduced holes of CdS can be oxidized by lactic acid and/or lignin as the hole scavengers. This consumption of holes in the valence band of CdS facilitates the minimization of charge recombination of the electron and hole; this resulted in the suppression of photocorrosion of CdS in the solution and ultimately improved the photocatalytic H_2_ production rate.

#### Carbon aerogels/CdS and regenerated cellulose/CdS

With renewable seaweed-derived carrageenan as the source of carbon and sulfur for CdS@Carbon Aerogels (CA/CdS), the hydrogen evolution rate was found to be 113.5 μmol h^−1^ g_cat_^−1^ with optimized carbon content for rapid photoelectron transfer and suppression of charge recombination [51]. Carrageenan was chosen for the carbon and sulfur donor for the preparation of CA/CdS. Regenerated cellulose films embedded with CdS NP in Na_2_S-Na_2_SO_3_ under visible light, in comparison with regular CdS, were proven to have improved radiative absorption in the 250–800 nm wavelength range and demonstrated increased photostability when compared to bare CdS nanoparticles. When loaded with Pt, the hydrogen production amounted to 1.323 mmol h^−1^ g_cat_^−1^ [52]. Interstitial P-doping of Zn_x_Cd_1-x_S to promote S-vacancies resulted in an H_2_ production rate of 419 μmol h^−1^ g^−1^_cat_, and its photocatalytic activity was explained by long-lived charge carriers and the increased formation of photo-hole pairs from the doping [50].

## Metal-free based photocatalysis

In pursuit of more green and sustainable chemistry for the generation of H_2_ from biomass, a variety of metal-free photocatalysts such as poly(p-phenylene), graphitic carbon nitride, boron carbides, and graphite oxide have been widely developed [53–58]. For example, graphitic carbon nitride (g-C_3_N_4_) was first explored for hydrogen production under visible light [59]. However, g-C_3_N_4_ has demonstrated limited photocatalytic efficiency on its own, due to low surface area, limited light absorption, and high rate of charge recombination [60]. In order to overcome these deficiencies through inorganic heteroatom doping without utilizing chemicals that compete with food, Jiang et al*.* realized a high-value application of crude bio-oil that circumvents expensive and industrially-infeasible preparation or upgrading, by doping g-C_3_N_4_ with non-purified bio-oil produced from pyrolyzed pine sawdust [61]. Insertion of C–O functional groups on the surface of the g-C_3_N_4_ through solvothermal treatment was observed by X-ray photoelectron spectroscopy to lower the conduction band for improved hole-separation and hydrogen reduction. A maximal hydrogen evolution rate of 1654 μmol h^−1^ g_cat_^−1^ was achieved by solvothermal treatment at 180 °C with 1:1 weight ratio of bio-oil to g-C_3_N_4_.

Aqueous room-temperature photoreforming of α-cellulose via cyanamide-functionalized carbon nitride, ^NCN^CN_x_, to H_2_ generation was optimized through addition of a molecular bis(diphosphine) Ni proton reduction co-catalyst to ^NCN^CN_x_. Under AM 1.5G irradiation, an H_2_ production yield of 2.62 μmol was achieved, with alkaline conditions allowing for optimal hydrogen production yields [4]. This was largely attributed to the ability of the functionalized cyanamide surface to transfer photoformed holes to electron donating substrates. This system was utilized furthermore on xylan and lignin and their respective moieties, where it was found that the limiting factor in hydrogen evolution was the presence or formation of smaller substrate molecules able to proceed to the surface of the catalyst to quench photoformed holes for hydrogen production. With sawdust as a raw biomass feed, 202 μmol h^−1^ g_cat_^−1^ of H_2_ was achieved. However, the proton reduction co-catalyst lacks structural stability and was demonstrated to degrade after 24 h, allowing other co-catalysts with slower kinetics to produce much greater amounts of hydrogen, outperforming even after 12 days.

Metal-free electrodes for hydrogen evolution by water splitting can be developed from biomass that would otherwise be utilized inefficiently as fossil fuels or consumed by pyrolysis. For hydrogen production, recent work reports the use of biomass products (e.g. char) as a metal-free carbon-based electrode pellets formed without binders that sacrificially decompose, and whose electrochemical performance can be modified through surface enhancement or exchange of electrolyte identity [62]. For example, Ding et al*.* doped the carbon-based electrode with nitrogen and demonstrated increased stability and similar resistivity as non-doped carbon-based electrodes [62]. Research such as this invokes a call for chemical modification in light of solar-driven hydrogen evolution as a system that ideally incorporates as much biomass as possible.

## Biomass-derived substrates

Photocatalytic water splitting to obtain hydrogen gas using low-energy ambient reaction conditions and solar radiation as the only energy input will contribute to the realization of future low-cost, environmentally-conscious fuels. As an alternative to water, biomass offers another possible source of electrons and protons for the photochemical formation of hydrogen, with the added possibility of forming value added organic products, depending on the anodic chemistry coupled to the cathodic H_2_ generation [5, 16, 18]. Effective photochemical biomass-to-hydrogen processes relying on solar energy inputs could valorize biomass feedstocks currently viewed as waste products, such as pulp generated during paper processing. Moreover, the possibility that this photochemical approach could also enable the production of low molecular weight aromatic products from the biomass stream concomitant with H_2_ production could offer another economic advantage [2]. The targeted and currently unused biomass sources, such as raw lignocellulosic biomass, do present difficulties such as limited solubility and chemical inertness [63]. These can be overcome with pre-processing methods, such as acid hydrolysis or ball-milling, but with added cost in the form of higher energy input needed for the overall reaction [64]. It must be noted that nutritionally relevant substrates (e.g. starch) whose use would compete with the food industry are not considered valid as a biomass source for H_2_ production. This section will review the range of raw biomass and biomass-derived substrates that do represent viable sources for the photocatalytic production of hydrogen gas. The consideration will go in order of molecular size, starting with monomeric substrates such as alcohols, aldehydes, carboxylic acids, and glucose, then moving onto saccharides and ending with raw lignocellulosic biomass itself. Reported efficiencies for the photocatalytic reaction and hydrogen yields will be considered, along with the difficulties or benefits of a given substrate type.

### Monomeric substrates

A myriad of monomeric waste products from industrial biomass processing could serve as feedstock for H_2_ production, including methanol from syngas production, ethanol from sugar fermentation, and glycerol from biofuel production [22, 65, 66]. However, it may be the case that some of these sources of industrial production are not relevant to the future of green chemistry. Ideal biomass-derived substrates for hydrogen production are those that are produced from industrial processes that are not energy intensive or based on fossil fuels. The most promising are categorized by functional groups of monomeric substrates including alcohols, acetaldehydes, amines, and carboxylic acids. Important metrics for the photocatalytic hydrogen evolution from these monomeric substrates in the presence of visible light are summarized in Table 1 and discussed in detail, along with molecules of the same functional groups that have research relevance as model compounds for mechanistic studies or test substrates. Indeed, many of these molecules will be transiently formed in the photo-reformation of higher-order oxygenates or polymeric substances, and thus conducting research of their action in hydrogen evolution aids in the understanding of overall biomass photo-reformation.

**Table 1:** Photocatalytic hydrogen production from monomeric substrates using visible light sources

Photocatalyst	Substrate	Power intensity mWcm^−2^	Production rates mmol h^−1^g_cat_^−1^	Refs.
Pt/TiO_2_/SiO_2_	MeOH/H_2_O	100 (AM 1.5G)	497	Han et al. 2015 [22]
Au/TiO_2_	MeOH/H_2_O	– (Solar Simulator)	1.4–7.0	Serra et al. 2015 [38]
Au/TiO_2_	EtOH	100 (Solar Simulator)	5–6	Puga et al. 2014 [35]
CuO_x_/TiO_2_	EtOH	100 (Solar Simulator equipped with 150 W Xe lamp)	– (4 mg h^−1^ g^−1^_cat_)	Ampelli et al. 2013 [67]
Fe_2_O_3_	EtOH	– (Solar Simulator)	– (20 mmol h^−1^ m^−2^)	Carraro et al. 2014 [68]
Ag/Fe_2_O_3_	EtOH	– (Solar Simulator)	– (24.0 mmol h^−1^ m^−2^)	Carraro et al. 2014 [68]
Au/Fe_2_O_3_	EtOH	– (Solar Simulator)	– (45.0 mmol h^−1^ m^−2^)	Carraro et al. 2014 [68]
Pt/TiO_2_-nanotubes	EtOH	– (Low-power solar lamp, 60 W tungsten)	– (37.1 μmol h^−1^ cm^−2^)	Ampelli et al*.* 2010 [69]
Cu_2_O/TiO_2_-nanorods	Glycerol	– (Natural sunlight)	50.339	Kumar et al.. 2015 [70]
TiO_2_-nanorods	Glycerol	– (Natural sunlight)	2.95	Kumar et al. 2015 [70]
CuO/TiO_2_-nanotubes	Glycerol	– (Natural sunlight)	99.823	Kumar et al. 2013 [71]
ZnO/ZnS-nanorods	Glycerol	– (500 W Xe)	0.3884	Sang et al. 2012 [72]
Cu_2_O-microcrystals	Formaldehyde	50 (Xe > 420 nm)	– (82.2 μmol in 3 h)	Gao et al. 2015 [73]
Pt@ZnIn_2_S_4_/RGO/BiVO_4_ (Z-scheme)	Formaldehyde	–	1.687	Zhu et al. 2019 [74]
Ir-Bpy-ENT (Iridium-based bipyridine- and ethenyl-incorporated bifunctional organosilica nanotubes)	Formaldehyde	– (Vis > 420 nm)	– (14.9 mL in 5 h)	Zhang et al. 2018 [75]
Cu/TiO_2_	Acetic acid	100 (AM 1.5 G)	0.036	Imizcoz et al. 2019 [76]
NiS/CdS	Lactic acid (with lignin)	– (300 W Xe $\ge$ 400 nm)	1.5124	Li et al. 2018 [50]
Pt/Holey carbon nitride-N-acetylethanolamine (HCN-NEA)	Triethanolamine	– (300 W Xe)	22.043	Liu et al. 2020 [77]
Poly(3-hexylthiophene)/g-C_3_N_4_	Ascorbic acid	334.8 (300 W Xe $\ge$ 500 nm)	− (3.045 μmol h^−1^)	Zhang et al. 2015 [78]
Poly(3-hexylthiophene)/g-C_3_N_4_	Ethylenediamine tetra-acetic acid	6.3 (300 W Xe $\ge$ 420 nm)	0.044	Zhang et al. 2015 [78]
Poly(3-hexylthiophene)/g-C_3_N_4_	Triethanolamine	6.3 (300 W Xe $\ge$ 500 nm)	− (0.104 μmol h^−1^)	Zhang et al. 2015 [78]
Pt/C_3_N_4_-TiO_2_	Triethanolamine	– (250 W visible light source)	1.042	Alcudia-Ramos et al. 2020 [79]
Mn-MOF@Au	Triethylamine	– (2.02 W white LED)	0.6	Luo et al. 2018 [80]

#### Alcohols

Methanol can be formed by the syngas process, but the energy intensive nature of this approach, and its general reliance on fossil fuels as a source of carbon, make this an unattractive source of methanol as part of a green-energy future [81]. Fermentation generates ethanol from biomass derived sugars [82], but the importance of ethanol as a fuel and the relevance of this process and the sugar sources to the food industry make this an unreasonable feedstock for the production of H_2_ [83]. However, the mechanistic studies using these alcohols for hydrogen evolution can provide important information on catalyst activity. Methanol in particular has been studied extensively in this regard [27, 32, 58, 84, 85].

**Fig. 9:**
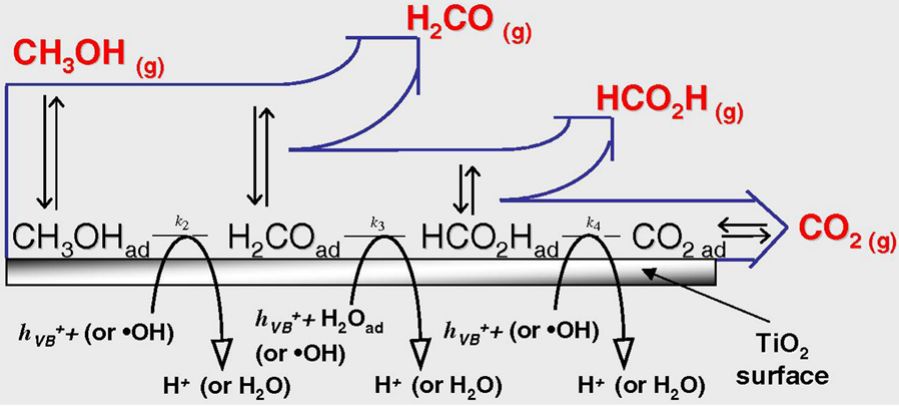
The photoreformation mechanism of methanol over TiO_2_ semiconducting material, featuring adsorption and desorption events and chemical transformations into formaldehyde, formic acid, CO_2,_ and H_2_. Reprinted with the permission from Chiarello et al. [86] (Copyright 2011 Elsevier)

The photo-reformation mechanism of methanol to H_2_ and CO_2_ observed using Pt/TiO_2_ or Au/TiO_2_ surfaces features cyclic adsorption and desorption processes for each reduction and subsequent oxidation step proceeding from methanol → formaldehyde → formic acid → H_2_ and CO_2_, as resolved by limiting the irradiation time and sampling the resulting solution [86]. However, CO has also been found in the reaction solution along with CH_4_, with decreasing concentrations when shifting from UV to visible irradiation [86]. CO can act to poison Pt islands on TiO_2_ catalysts [23], especially at low loading concentrations. It has been rationalized that CO absorption onto Pt/TiO_2_ photocatalysts induces oxygen vacancies that deactivate the noble metal active sites. Thus, an optimized loading weight of 1 wt% noble metal onto the semiconducting material has been found for methanol photo-reforming [84]. It is generally accepted that the methanol photo-reformation pathway occurs in the consolidated mechanism demonstrated in Fig. 9, though more emphasis on the composition of the reaction mixture during experimentation would help clarify this mechanism. Studies in this field tend to focus on hydrogen evolution enhancement and not on compositional analysis, but better analysis of the resulting composition of the reaction mixtures would aid elucidation of the mechanism for a given catalyst.

Ethanol generation is a well-founded source of biofuels through hydrolysis of lignocellulosic biomass [87]. Studying it as a substrate for photochemical hydrogen production is important as it is a model of the primary alcohol groups found in biomass. Dehydrogenation of ethanol produces stoichiometric equivalents of hydrogen gas and acetaldehyde, rationalized through a two-electron transfer step using Au/TiO_2_ as the photocatalyst. However, the expected subsequent oxidation step is hindered by inadequate adsorption of the acetaldehyde onto the photocatalyst surface; instead, side-reactions producing the hemiacetal 1-ethoxyethanol have been observed [35]. The presence of another carbon atom increases the complexity and diversity of photo-reforming pathways of the alcohol.

The complete photo-reformation of ethanol into hydrogen and carbon dioxide gas proceeds with hydrogen gas first formed in the reaction sequence following dehydrogenation to give acetaldehyde [88]. Carbon dioxide subsequently forms during the proceeding oxidation steps. Side reactions include the formation of C–C bonds by the 1-hydroxyethyl radical species produced following the first single-electron transfer to the catalyst. Controlling the relative surface area of the TiO_2_ in the Pt/TiO_2_ construct can suppress this reaction [89]. This was rationalized by higher TiO_2_ surface areas promoting adsorption of substrate intermediates, increasing the likelihood of oxidation reactions as opposed to radical coupling interactions. From these and other observations, Bahruji et al*.* determined that the ability to photo-reform a variety of alcohols, C_x_H_y_OH, using a Pd/TiO_2_ photocatalyst to hydrogen and hydrocarbon C_x-1_ products depended on a set of general rules: (1) the availability of hydrogen on the C_α_–OH, (2) the presence of alkyl groups attached to the alcohol, (3) the presence of methylene groups between alcohol functional groups, and (4) the relative strength of the alpha C–C bond [90].

**Fig. 10:**
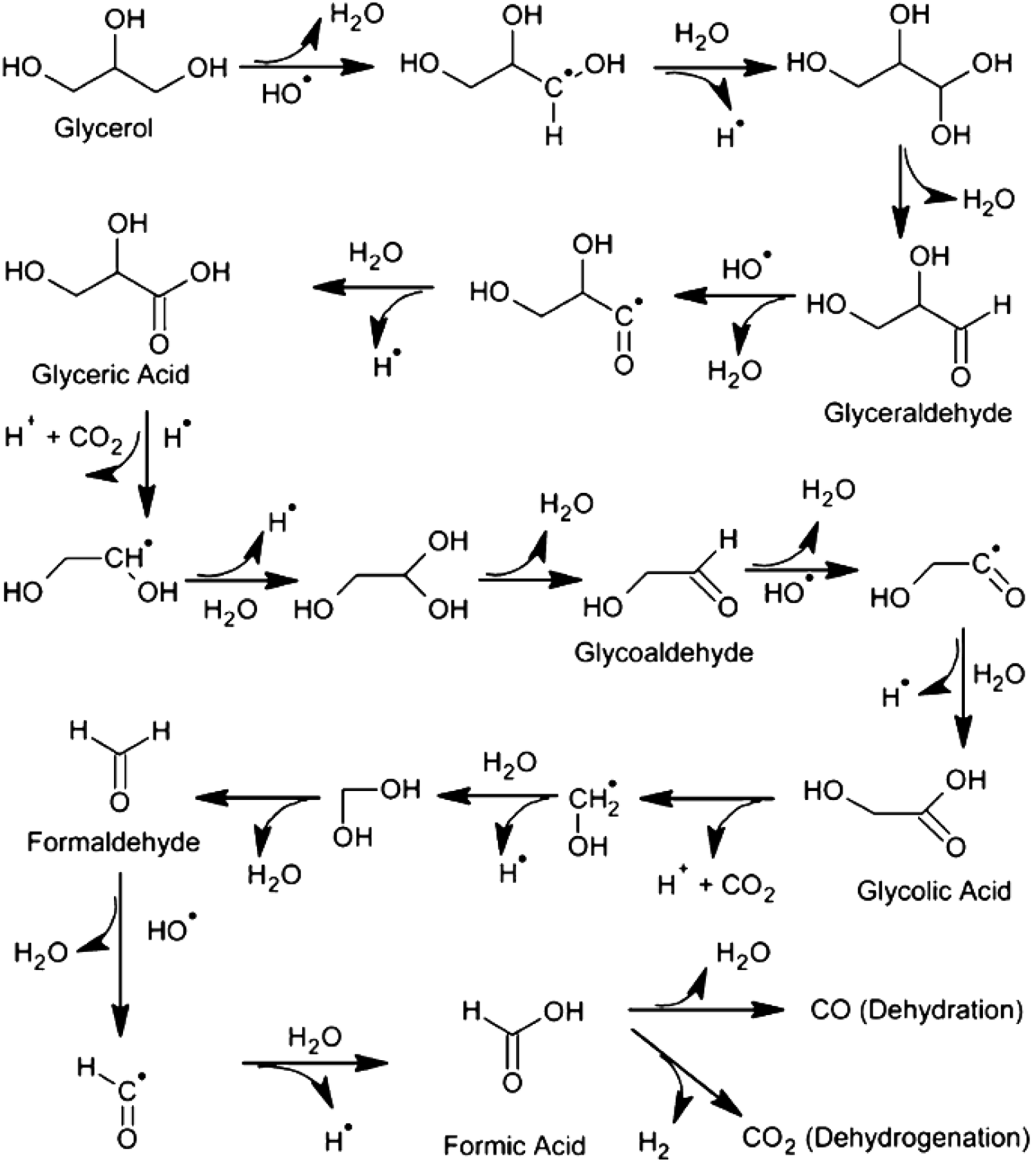
Photoreformation pathway for hydrogen production from glycerol biomass derivative. Reprinted with the permission from Puga et al. [5] (Copyright 2016 Elsevier)

Glycerol as a biomass product is the most relevant alcohol of study due to its major production in biofuel industry [91]. Although methanol and ethanol facilitate research progress in this field, glycerol is a current biomass waste product with low market value and thus is an ideal candidate for chemical valorization. Moreover, it sits in the sweet-spot of a higher-order oxygenate without structural complexity and can bridge the development of photocatalytic systems for more complex biomass substrate. Isotopic experiments similar to those described for methanol were performed with glycerol resulting on the contrary observation that the proton source with this polyol is actually from its three-fold hydroxyl groups instead of water [92]. Photo-oxidation (4) and photo-reforming reactions (5) of glycerol have been investigated using irradiated Pt/TiO_2_ (Fig. 10) [93]. Initial photooxidation steps involve the hydrogenolysis of glycerol to propylene glycol and dehydration of glycerol to glyceraldehyde. Then, subsequent reactions such as dehydration, dehydrogenation, and decarbonylation allow the formation of various reaction intermediates including acetaldehyde, ethanol, acetol, and methanol. In the above reactions, hydrogen and CO are readily oxidized by photogenerated oxidant species to H_2_O and CO_2_ under the gas-phase O_2_ atmosphere. However, during the photo-reforming reaction process, hydrogen is produced in the gas phase followed by desorbing hydrogen from the irradiated photocatalyst surface.

4 $${\text{C}}_{{3}} {\text{H}}_{{8}} {\text{O}}_{{3}} + \, \left( {{7}/{2}} \right){\text{O}}_{{2}} \to {\text{ 3CO}}_{{2}} + {\text{ 4H}}_{{2}} {\text{O}}$$

5 $${\text{C}}_{{3}} {\text{H}}_{{8}} {\text{O}}_{{3}} + {\text{ 3H}}_{{2}} {\text{O }} \to {\text{ 3CO}}_{{2}} + {\text{ 7H}}_{{2}}$$

Over a Cu/TiO_2_ photocatalyst, the production of hydrogen has been investigated with intermediate formation of acetone, acetaldehyde, and formic acid by reforming glycerol [94, 95]. The complexity of the resulting reaction mixture composition has generated questions about whether polyols or monoalcohols are more efficient in photocatalytic hydrogen evolution and whether the structural isolation or adjacency of the polyol hydroxyl groups have an effect on the efficiency. It has been reported that hydroxyl groups separated by aliphatic segments tend to yield alkane products, while the grouping of hydroxyl groups within a compound (as is found in higher oxygenated biomass-derived substrate) are indeed more efficient in the photocatalytic hydrogen evolution reaction at low concentrations. A study of Ni and Au augmented TiO_2_ photocatalysts suggested that the polarity and oxidation potential of different alcoholic substrates are directly related to the achievable hydrogen production rates [96]. Other reports however have shown that the reaction kinetics depend on the entire photocatalytic set up not just the identity of the alcohol substrate [85]. These results call for further research in order to provide a more definite conclusion on alcohol photoreactivity within a system developed for hydrogen evolution.

#### Aldehydes

Aldehydes are common intermediates formed during biomass reformation, and formaldehyde as the simplest aldehyde represents a key substrate for use in understanding the photo-reformation pathways of this class of molecules. One equivalent each of formaldehyde and water can be photochemically reformed to give two equivalents of molecular hydrogen and one of carbon dioxide. This process typically involves the initial oxidation of formaldehyde and reaction with water to give formic acid and the subsequent reduction of the H^+^ equivalents H_2_. It has recently been demonstrated in a Z scheme ZnIn_2_S_4_/RGO/BiVO_4_ system for hydrogen evolution that the capacity for aldehydes, and in particular formaldehyde with the fewest carbon atoms, to form enol anions allows them to be efficient hole-trapping substrates in comparison to alcohols and organic acids [74]. However, formaldehyde-based photocatalytic systems outside of that particular scheme are largely reported to have one- or two-fold lower hydrogen yield than alcohol-based systems [97]. High photochemical hydrogen gas production rates have been reported using formaldehyde as reductant with solitary simple oxides such as Cu_2_O microcrystals [73]. Such an observation can be rationalized by a unique adsorption characteristic of formaldehyde onto the surface of the photocatalyst which promotes the oxidation half-reaction [73, 98]. Acetaldehyde, mentioned as an intermediate in the ethanol photo-reformation pathway above, gives similar lower reaction yields as found for formaldehyde in comparison to alcohols [99]. In this case stable adsorption onto the photocatalyst surface by the carboxyl groups present might also play a role but, under the reaction conditions, by decreasing the interaction of the surface-bound aldehyde with radical species in solution. The same trend was observed with glyceraldehyde, an intermediate in the photo-reformation of glycerol [97]. More recently, a molecular based photocatalyst system was demonstrated to evolve 14.9 mL of hydrogen using an aldehyde substrate (with 100% of the gas collected being hydrogen itself) over an ethenyl-bridged organosilica nanotubes containing bipyridine ligands metalated with iridium (Ir-BPy-ENT) photocatalyst [75].

#### Amines

Recently, amines have been given attention as a potential biomass-derived substrate, specifically trimethylamine (TMA), triethylamine (TEA), triethanolamine (TEOA), and ethylendiamine tetraaceticacid (EDTA) [78, 80, 100]. A polymer–polymer surface heterojunction catalyst compared TEOA and EDTA in the same photocatalytic experimental parameters and found that the former outperformed the latter with double the hydrogen yield (104 to 44 μmol/h, respectively) [78]. This difference was rationalized not by the oxidation potentials of the substrates, but rather due to the oxidative mechanisms allowed and ultimately determined by the relationship between the polymer (e.g., poly(3-hexylthiophene))-liganded g-C_3_N_4_ photocatalyst. Luo et. al. reported a hydrogen production rate of 600 μmol h^−1^ g_cat_^−1^ was achieved over a Mn-MOF@Au photocatalyst using an aqueous triethyl-amine (TEA) containing solution as the source of electron [80]. In addition, work performed with TEOA over TiO_2_ and C_3_N_4_ revealed that this substrate was more photoactive on TiO_2_, suggesting that the photomechanism proceeds via the electron density located on the N atom of the amine group [100], although more details of the mechanistic pathways have not been investigated. When TEOA is used as a sacrificial hole scavenger over an N-acetylethanol-amine modified carbon nitride photocatalyst, a remarkable hydrogen evolution rate of 22,043 μmol h^−1^ g_cat_^−1^ is achieved [77], which far exceeds a similar unmodified g-C_3_N_4_/TiO_2_ photocatalyst which achieved 1041 μmol h^−1^ g_cat_^−1^ by comparison [79].

#### Carboxylic acids

In addition to the formation of carboxylic acids in the photoreforming of biomass, this moiety can also be produced by the industrial fermentation of saccharides [82]. A major component of lignocellulosic biomass, hemicellulose, is rich in ester-containing moieties that are comprised of monomeric carboxylic acid compounds. During photoreformation, carboxylic acid substrates are able to bind tightly to their metal-oxide catalyst counterparts (e.g. TiO_2_) through hydroxyl and carbonyl functional groups [101, 102], which can have mixed results relating to the production of hydrogen.

Lactic, acetic, and formic acid are the principally studied biomass-derived carboxylic acid substrates due to their formation during biomass-processing such as fermentation [82]. The selective use of photocatalyst materials can determine whether these compounds evolve via dehydrogenation of the hydroxyl group or decarboxylation of the carbonyl group. For example, with platinum as co-catalyst, CdS was demonstrated to carry out the dehydrogenation of lactic acid with minor CO_2_ generation, while TiO_2_ preferred the decarboxylation step; this was realized by the CdS formation of pyruvic acid, and the TiO_2_ formation of acetaldehyde, acetic acid, ethanol, and primarily CO_2_ [103]. The difference in selectivity has been rationalized by the potential difference in band energy positions and surface adsorption modes. It is clear that, despite the greater efficiency of CdS photoreformation, TiO_2_ would be preferred for complete photoreformation of carboxylic acid substrates due to its ability to react with subsequently produced photo-substrates. Recently, a new photocatalyst CdS@g-C_3_N_4_ was able to convert lactic acid into H_2_ at a maximum rate of 19.88 mmol h^−1^ g_cat_^−1^ without the use of noble metal catalyst [104]. It was found that efficient charge separation was determined not by morphology of the semiconductor, but rather of the sulfur vacancies introduced by the graphitic carbon nitride. By increasing the sulfur vacancies of different variants of the CdS@g-C_3_N_4_ photocatalysts, the high hydrogen production rate was demonstrated.

Recently, a method of favoring the photoreformation pathway over the decarboxylation pathway into CH_4_ was demonstrated over Cu/TiO_2_ catalyst by controlling the oxidation state of Cu islands on the photocatalyst [76]. Over the duration of uncontrolled photoreformation of acetic acid, these islands became oxidized to CuO, which favors decarboxylation as opposed to H_2_ production (Eqs. 6, 7). By in situ photodeposition of the Cu into the reaction mixture after solar reformation, the H_2_/CH_4_ ratio was increased two-fold, and the retention of surface copper reduction sites Cu(0) improved.

6 $${\text{CH}}_{{3}} {\text{COOH}}_{{({\text{l}})}} \to {\text{CH}}_{{{4}({\text{g}})}} + {\text{ CO}}_{{{2}({\text{g}})}}$$

7 $${\text{CH}}_{{3}} {\text{COOH}}_{{({\text{l}})}} + {\text{ 2H}}_{{2}} {\text{O}}_{{({\text{l}})}} \to {\text{2CO}}_{{{2}({\text{g}})}} + {\text{ 4H}}_{{{2}({\text{g}})}}$$

In addition, formic acid as a substrate has been demonstrated to decompose into CO as well as CO_2_ and H_2_ by chalcogenides or under UV-rich light, requiring greater judicious efforts when selecting a Pt/CdS photocatalyst for a system in which CO is not readily able to be abstracted or is detrimental to the photoreactive system [105]. Moreover, a recent study that investigated formic acid and methanol as sacrificial electron donors in hydrogen evolving water-splitting photocatalysis over several metals (Au, Cu, Pt, Co, Ru, Pd)@TiO_2_ semiconductors found that, although formic acid outperformed methanol for the production of hydrogen in five of the seven photocatalytic systems (up to 22.8 mmol h^−1^ g_cat_^−1^ over Au@TiO_2_), that these improved rates were negligible at low concentrations of substrate (1%) [37]. Interestingly, the authors suggested that the use of sacrificial electron donors to improve the hydrogen production rates of photocatalytic water splitting should be considered carefully, as their improvement of the reaction varies greatly with reaction conditions such as pH and type of photocatalytic materials used. Beyond their use for mechanistic studies, sacrificial donors should be avoided unless sourced from waste streams as these same chemicals often have commercial or industrial value. An example of a potential source of sacrificial donor stream is the used dairy and brewery waste effluent in the photocatalytic experiments of Speltini et al., whose results are demonstrated shown in Table 2 [106].

**Table 2:** Photocatalytic hydrogen production from saccharides using visible light sources

Photocatalyst	Saccharides	Power intensity	Production rates mmol h^−1^ g_cat_^−1^	Refs.
		mW cm^−2^		
Pt/TiO_2_	Glucose	300 (Solar simulator)	2.4	Kondarides et al. 2010 [107]
Pt/Holey carbon nitride-N-acetylethanolamine (HCN-NEA)	Glucose	– (300 W Xe > 420 nm)	5.5808	Liu et al. 2020 [77]
Au/CdS-nanorods	Glucose	– (300 W Xe > 400 nm)	0.09	Wang et al. 2020 [8]
Pt/TiO_2_	Galactose	– (450 W Xe)	– (*ca.* 5.5 µmol min^−1^)	Kondarides et al. 2008 [9]
HCN-NEA	Galactose	– (300 W Xe > 420 nm)	– (156.3 µmol h^−1^)	Liu et al. 2020 [77]
Pt/TiO_2_	Mannose	300 (Xe solar)	1.725	Kondarides et al. 2010 [107]
Pt/TiO_2_	Ribose	300 (Xe solar)	2.175	Kondarides et al. 2010 [107]
Pt/TiO_2_	Lactose	– (450 W Xe)	– (4 µmol min^−1^)	Kondarides et al. 2010 [107]
HCN-NEA	Cellobiose	– (300 W Xe > 420 nm)	– (75.6 µmol h^−1^)	Liu et al. 2020 [77]
Pt/TiO_2_	Cellulose	– (Natural Sunlight)	– (33 µmol over 4 h)	Speltini et al. 2014 [17]
TiO_2_/NiO_x_@C_g_	Cellulose	– (500 W Xe)	0.27	Zhang et al. 2018 [42]
Pt/TiO_2_	Brewery/Dairy effluent	50 (Solar Simulator)	*ca.* 0.286/*ca.* 0.00	Speltini et al. 2019 [106]
Cu-Ni/TiO_2_	Brewery/Dairy effluent	50 (Solar Simulator)	*ca.* 0.150/*ca.* 0.080	Speltini et al. 2019 [106]
Cu-Ni/o–g-C_3_N_4_	Brewery/Dairy effluent	50 (Solar Simulator)	*ca.* 0.040/*ca.* 0.035	Speltini et al. 2019 [106]
Pt/o–g-C_3_N_4_	Brewery/Dairy effluent	50 (Solar Simulator)	*ca.* 0.120/*ca.* 0.110	Speltini et al. 2019 [106]

### Saccharides

The formation of mono-, di-, and polysaccharides when photocatalyzing raw biomass makes saccharide-based studies widely applicable to other biomass systems of photocatalysis [8, 77, 107, 108]. Photocatalytic hydrogen production from saccharides under visible light illumination is summarized in Table 2. When comparing groups of saccharides, it is found that an increase in structural complexity leads to a decrease in hydrogen evolution. For example, over carbon nanotube modified Pt/TiO_2_, decreasing hydrogen evolution was found in order of arabinose > glucose > fructose > cellobiose as substrates [109]. Recently, a polymeric carbon nitride formed from curly-like carbon nitride nanosheets with in-plane surface dyadic heterostructure was proven an efficient hydrogen generation catalyst for the conversion of a variety of saccharides, including mono- and disaccharides, hemicellulose, and cellulose [77]. Regardless of photocatalytic material, the solubility of the saccharide determined its ability as a photocatalytic substrate in hydrogen evolving aqueous mixtures [63]. By obtaining saccharide-sources that are already solubilized into waste streams from dairy and brewery effluents, Speltini et al. demonstrated efficient conversion into hydrogen over four separate photocatalytic systems, with the brewery effluent over Pt/TiO_2_ achieving as much as 286 μmol h^−1^ g_cat_^−1^ [106].

#### Glucose

Glucose as a model biomass-derived substrate has been extensively studied for photocatalytic production of hydrogen gas due to its prevalence in biological systems and higher-order biomass structure [8, 39, 108]. The smaller molecular size and increased presence of abstractable hydrogens confer greater solubility, and glucose generally shows increased activity compared to higher-order saccharides in identical systems [77]. For example, different noble-metal-loaded TiO_2_ photocatalysts were used to generate hydrogen gas economically and effectively from glucose [39]. Other monosaccharides have been studied—including hexoses (fructose, galactose, and mannose) and pentoses (xylose, arabinose, and ribose)—but not as extensively as glucose and show similar hydrogen production capabilities when acting as biomass-derived substrates for photocatalytic hydrogen production [5].

#### Cellulose/cellobiose

The production of hydrogen gas by photocatalyzed consumption of cellulose as a sacrificial electron donator is a popular research topic, due to the fact that cellulose is a major component of lignocellulosic biomass [17, 42]. Currently, H_2_ can be produced from cellulose by thermochemical gasification and acid hydrolysis [3], but as these methods are less energy efficient than photocatalytic methods, research has shifted focus towards photocatalysis. Zou et al. demonstrated that combining methods of acid hydrolysis and photocatalytic water splitting can overcome cellulose insolubility [25]. By hydrolyzing cellulose into glucose, an increase in carbohydrate solubility can be obtained, thereby increasing the photocatalytic production of hydrogen five-fold (*ca.* 33 μmol without cellulose hydrolysis, to 170 μmol with cellulose hydrolysis), without affecting the photocatalyst stability. This method was even demonstrated on 500 mg of paper pulp, whereby the hydrogen production increased to 1320 μmol h^−1^ g_cat_^−1^, but the fact remains that this method is hindered by the reaction conditions (i.e. 403 K and 0.6 M sulfuric acid) necessary to stimulate acid hydrolysis of cellulose in the system. Another method to overcome cellulose insolubility is the use of ball-milling and immobilization pretreatment of cellulose to increase surface area exposure between cellulose and the photocatalyst, demonstrated by Zhang et al. By mechanochemically converting the crystalline cellulose into submicron sizes (0.1–3 μm) and immobilizing it onto photocatalytic Pt/TiO_2_ nanoparticles, they achieved enhanced hydrogen production over non-immobilized controls. A systemic study found that phototransformation of cellulose and water to hydrogen gas could occur in mineralized seawater and pH 7.4 conditions gave the highest yield of hydrogen [17]. Unexpectedly, it was also observed that HMF production causes an *in-situ* dye-sensitization of the photocatalyst by absorption of HMF oligomers, as its photoreforming is less favored than other carbohydrate-substrates formed during the reaction. This effect increases the absorption in the visible region of the photocatalyst, an additive effect of an already expanded visible-range absorption by Pt on TiO_2_.

Disaccharides such as cellobiose produced from the hydrolysis of cellulose perform better than their parent species for hydrogen production [106], but still inherit the encumbering structural complexity that limits its efficiency in the photocatalytic reaction system. Despite this quality, the disaccharides lactose and sucrose have been shown to undergo complete conversion to H_2_ and CO_2_ upon phototransformation [110]. Hydrogen gas production rates *ca.* 3000 μmol/h/g_cat_ have been demonstrated with sucrose, lactose, and cellobiose [107].

## Raw biomass

The focus of this review is on the ultimate use of raw biomass as the source of protons and electrons for the photocatalytic production of hydrogen production. The capacity for humans to take waste byproducts from current biomass industrial utilization and valorize them into hydrogen fuel and low molecular weight chemicals could play a key part in more sustainable economy by reducing reliance on fossil fuels and enabling more complete use of appropriated biomass. The chemical and structural complexity of raw biomass limits its use in photocatalytic applications and requires the implementation of pretreatment methods before lignocellulosic valorization, which in and of itself is an active field of current research [111]. Moreover, the low solubility of biomass inhibits the photocatalytic hydrogen production process [63]. While pretreatment methods add to the cost of the overall process, they are currently necessary to achieve high hydrogen yields [112]. Despite these drawbacks, research has been performed over varied raw biomass products [113, 114], ranging from agricultural waste to dead insects [115], with Pt/TiO_2_ being the most popular and effective choice of photocatalyst. It was found that aquatic biomass, such as seaweed, enabled higher efficiencies than land-derived biomass, presumably due to the lack of lignin necessary to maintain their biological rigidity and the limited UV penetration at the depth of water they reside [63]. Considering plant-derived biomass, Table 3 summarizes photocatalytic studies utilizing biomass mostly without pretreatment for the production of H_2_. For example, the production rate of 0.061 mmol h^−1^ g_cat_^−1^ was achieved with fescue grass over Pt/TiO_2_ substrate by elevating the reaction temperature to 60 °C [116]. Wakerly et al*.* were able to demonstrate that hydrogen evolution varies widely over different selected biomass substrates, but that all were enhanced by the alkalinity of the solution and thus the solubility of the substrate [114]. A systemic study found that rice husk demonstrated a hydrogen production rate of 0.095 mmol/g/h in a simple photocatalytic setup of water and sunlight. For the sake of comparison, the last two entries in Table 3 include pretreatment methods: phosphoric acid and natural degradation/liquid filtration of wheat straw and wastepaper scraps, respectively. A cyanamide-functionalized carbon nitride photocatalyst was able to achieve nearly a quarter of the hydrogen production rate in KP_i_ solution [4] in comparison to a visible-light sensitive Co/CdS/CdO_x_ photocatalyst in 10 M KOH solution [114], demonstrating the versatility of different methods in attempting to achieve the same goal.

**Table 3:** Photocatalytic hydrogen production from raw biomass using visible light sources

Photocatalyst	Substrate	Power intensity mW cm^−2^	Production rates mmol h^−1^ g_cat_^−1^	Refs.
Pt/TiO_2_	Fescue grass	– (150 W Xe)	0.061	Caravaca et al. 2016 [116]
Co/CdS/CdO_x_	Wooden branch	100 (AM 1.5 G)	5.31	Wakerley et al. 2017 [114]
Co/CdS/CdO_x_	Sawdust	100 (AM 1.5 G)	0.75	Wakerley et al. 2017 [114]
Co/CdS/CdO_x_	Grass	100 (AM 1.5 G)	1.0	Wakerley et al. 2017 [114]
Co/CdS/CdO_x_	Bagasse	100 (AM 1.5 G)	0.37	Wakerley et al. 2017 [114]
Cyanamide-functionalized carbon nitride (^NCN^CNx)	Sawdust	100 (AM 1.5 G)	0.202	Kasap et al. 2018 [4]
CoO/g-C_3_N_4_	Wheat straw^a^	– (300 W Xe)	129 µmol g_cat_^−1^	Wu et al. 2020 [20]
Au/CdS	Waste paper scraps^a^	– (300 W Xe > 400 nm)	0.0276	Wang et al. 2020 [8]

^a^These studies did pretreat the biomass substrate prior to photocatalytic utilization

## Conclusions and outlooks

Although progress has been made in regard to the photocatalytic reforming of biomass into value-added chemical substrates and molecular hydrogen, much research is needed to find active photocatalysts composed of low-cost, non-toxic materials that can sustain high rates of H_2_ production without the need for pretreatment of the biomass source. Metal oxide semiconductors and noble metal catalysts have established a starting point in this research, but the use of metal free semiconductor materials such as C_3_N_4_-based systems is desirable as these materials can be regenerated more effectively and economically from renewable resources. In the past, this research field has mainly focused on light absorbing semiconductors that depended on the absorption of UV light to drive photocatalytic activity. Recent focus has shifted to designing narrow band-gap materials and/or metal-free photocatalysts for H_2_ production from biomass to improve photocatalytic performance under visible or natural sunlight and the use of mild conditions to enhance selectivity toward value-added products along with H_2_ productivity.

Furthermore, the efficiency of this process needs to be taken into consideration, as it will be a balance between renewable semiconducting resources and the efficiency that will be sacrificed to make this composite possible. The use of biomass itself, not pre-refined biomass-derived molecules, as the electron-donating substrate in the photocatalytic generation of hydrogen gas is crucial to the realization of an industrially and environmentally relevant process. In regard to foundational mechanistic studies, much is still to be desired, and the results of these future studies will determine the absolute optimal reaction conditions to photo-reform biomass into specific platform molecules or for biofuel applications.

Lastly, photocatalysis offers the potential to replace the energy intensive approach of thermally reforming biomass for hydrogen production using abundant and free solar energy. Developing strategies using earth-abundant elements to form the light absorbers and photocatalysts for the capture and conversion of solar energy to electric potential energy, and eventually chemical potential energy in the form of the H–H bond, presents a key challenge in this field. As a carbon-free, energy-dense material, hydrogen will likely play an important role in mankind’s energy economy of the future. A method of supplying H_2_ in a sustainable, climate-neutral way must be developed, and solar-driven photochemical biomass conversion presents an obvious possible solution.

## Data Availability

Not applicable.
